# Dataset for file fragment classification of audio file formats

**DOI:** 10.1186/s13104-019-4856-1

**Published:** 2019-12-21

**Authors:** Atieh Khodadadi, Mehdi Teimouri

**Affiliations:** Information Theory and Coding Laboratory, University of Tehran, Tehran, Iran

**Keywords:** Audio file formats, Classification, File formats, File fragments

## Abstract

**Objectives:**

File fragment classification of audio file formats is a topic of interest in network forensics. There are a few publicly available datasets of files with audio formats. Therewith, there is no public dataset for file fragments of audio file formats. So, a big research challenge in file fragment classification of audio file formats is to compare the performance of the developed methods over the same datasets.

**Data description:**

In this study, we present a dataset that contains file fragments of 20 audio file formats: AMR, AMR-WB, AAC, AIFF, CVSD, FLAC, GSM-FR, iLBC, Microsoft ADPCM, MP3, PCM, WMA, A-Law, µ-Law, G.726, G.729, Microsoft GSM, OGG Vorbis, OPUS, and SPEEX. Corresponding to each format, the dataset contains the file fragments of audio files with different compression settings. For each pair of file format and compression setting, 210 file fragments are provided. Totally, the dataset contains 20,160 file fragments.

## Objective

A considerable amount of Internet traffic is used for exchanging audio file formats. As the sizes of these files are usually much bigger than the maximum network packet size, the files are segmented into fragments. The fragments generated by various users are transmitted over the network. Some of these fragments can be received by the network surveillance unit. The network surveillance unit may wish to detect the file format of each fragment for network forensics purposes.

Some researches have been carried in the field of file fragment classification of audio file formats [1–4]. There are a few publicly available datasets of files with different formats [5–7]. Therewith, there is no public dataset for file fragments of audio file formats. This makes it difficult for other researchers to compare the proposed methods with the existing methods.

In this study, we present a dataset that contains file fragments of 20 audio file formats: Adaptive Multi-Rate (AMR), Adaptive Multi-Rate Wideband (AMR-WB), Advanced Audio Coding (AAC), Audio Interchange File Format (AIFF), Continuously Variable Slope Delta modulation (CVSD), Free Lossless Audio Codec (FLAC), Global System for Mobile Communications Full Rate (GSM-FR), Internet Low Bitrate Codec (iLBC), Microsoft Adaptive Differential Pulse Code Modulation (ADPCM), MPEG Audio Layer-3 (MP3), Pulse-Code Modulation (PCM); Windows Media Audio (WMA), A-Law, µ-Law, G.726, G.729, Microsoft GSM, OGG Vorbis, OPUS, and SPEEX. Corresponding to each format, the dataset contains the file fragments of audio files with different compression settings.

## Data description

First, the whole set of the uncoded (raw) dataset of speech files is taken from www.voxforge.org [8]. These raw files are then converted in order to obtain audio files in 20 different formats: AMR, AMR-WB, AAC, AIFF, CVSD, FLAC, GSM-FR, iLBC, Microsoft ADPCM, MP3, PCM, WMA, A-Law, µ-Law, G.726, G.729, Microsoft GSM, OGG Vorbis, OPUS, and SPEEX. For each audio file format, different compression settings are considered. The raw data for all compression settings of a specific format is the same. However, there is no overlap between the raw data used for different formats.

96 pairs of file format and compression setting are considered. For each pair of file format and compression setting, we have 210 compressed audios. So, totally we have 20,160 audio files. Each of these files is segmented into 1 Kbyte (i.e. 1024 bytes) fragments. Then, one fragment is randomly selected among the fragments of each file. Before randomly selecting the fragments, 12.5% of the initial fragments and 12.5% of the final fragments of each file are discarded. This is to ensure that the fragments do not contain the file headers or trailers.

For each pair of file format and compression setting, we have 210 file fragments. So, the dataset of file fragments contains 20,160 file fragments. The dataset is partitioned according to 20 different file formats. Each partition is represented by an individual data file shown in Table 1. For example, data file 1 (i.e. aac.zip) contains 7 sub data files: aac-8.dat, aac-16.dat, aac-32.dat, aac-48.dat, aac-64.dat, aac-80.dat, and aac-96.dat. Sub data files are provided in a generic binary data file format with .dat file extension. Each sub data file contains 210 fragments.

**Table 1:** Overview of data files/data files

Label	Name of data file/data file	File types (file extension)	Data repository (DOI)
Data file 1	aac	Archive file format (.zip) containing multiple generic binary data (.dat) files	OSF (10.17605/OSF.IO/AHCYU)
Data file 2	adpcm	Archive file format (.zip) containing multiple generic binary data (.dat) files	OSF (10.17605/OSF.IO/AHCYU)
Data file 3	aiff	Archive file format (.zip) containing multiple generic binary data (.dat) files	OSF (10.17605/OSF.IO/AHCYU)
Data file 4	alaw	Archive file format (.zip) containing multiple generic binary data (.dat) files	OSF (10.17605/OSF.IO/AHCYU)
Data file 5	amr	Archive file format (.zip) containing multiple generic binary data (.dat) files	OSF (10.17605/OSF.IO/AHCYU)
Data file 6	awb	Archive file format (.zip) containing multiple generic binary data (.dat) files	OSF (10.17605/OSF.IO/AHCYU)
Data file 7	cvsd	Archive file format (.zip) containing multiple generic binary data (.dat) files	OSF (10.17605/OSF.IO/AHCYU)
Data file 8	flac	Archive file format (.zip) containing multiple generic binary data (.dat) files	OSF (10.17605/OSF.IO/AHCYU)
Data file 9	g726	Archive file format (.zip) containing one generic binary data (.dat) file	OSF (10.17605/OSF.IO/AHCYU)
Data file 10	g729	Archive file format (.zip) containing one generic binary data (.dat) file	OSF (10.17605/OSF.IO/AHCYU)
Data file 11	gsm	Archive file format (.zip) containing one generic binary data (.dat) file	OSF (10.17605/OSF.IO/AHCYU)
Data file 12	gsmwav	Archive file format (.zip) containing one generic binary data (.dat) file	OSF (10.17605/OSF.IO/AHCYU)
Data file 13	ilbc	Archive file format (.zip) containing multiple generic binary data (.dat) files	OSF (10.17605/OSF.IO/AHCYU)
Data file 14	mp3	Archive file format (.zip) containing multiple generic binary data (.dat) files	OSF (10.17605/OSF.IO/AHCYU)
Data file 15	ogg	Archive file format (.zip) containing multiple generic binary data (.dat) files	OSF (10.17605/OSF.IO/AHCYU)
Data file 16	opus	Archive file format (.zip) containing multiple generic binary data (.dat) files	OSF (10.17605/OSF.IO/AHCYU)
Data file 17	pcm	Archive file format (.zip) containing multiple generic binary data (.dat) files	OSF (10.17605/OSF.IO/AHCYU)
Data file 18	speex	Archive file format (.zip) containing multiple generic binary data (.dat) files	OSF (10.17605/OSF.IO/AHCYU)
Data file 19	ulaw	Archive file format (.zip) containing multiple generic binary data (.dat) files	OSF (10.17605/OSF.IO/AHCYU)
Data file 20	wma	Archive file format (.zip) containing multiple generic binary data (.dat) files	OSF (10.17605/OSF.IO/AHCYU)
Data file 21	SettingsTable	Portable document format (.pdf)	OSF (10.17605/OSF.IO/AHCYU)
Data file 22	ConversionSettings	Archive file format (.zip) containing 97 portable network graphics (.png) files	OSF (10.17605/OSF.IO/AHCYU)
Data file 23	ReadFragments	Matlab script file (.m)	OSF (10.17605/OSF.IO/AHCYU)

Data file 21 (i.e. SettingsTable.pdf) contains a table that specifies 96 pairs of file format and compression setting. In this table, the software program employed for generating each file format is also specified. Data file 22 (i.e. ConversionSettings.zip) contains several screenshots of the software programs that display the employed compression settings. Data file 23 (i.e. ReadFragments.m) is a script in MATLAB language that reads all the fragments from one or more sub data files. By running this script and selecting some sub data files, the fragments contained in these sub data files are read and stored in a variable name Dataset. Variable Dataset is a MATLAB cell array with two rows. Each column in this cell array corresponds to one of the selected sub data files. The first element of each column is a string value that specifies the sub data file name. The second element of each column is a structure array with only one field named fragments. Dataset {2, i}(j).fragments (j = 1,2,…,210) is a cell array with length one that contains one fragment of the jth file in the selected sub data file i.

## Limitations

- The size of the fragments is considered to be fixed and equal to 1024 bytes.
- A defined subset of file formats and compression settings are considered.

## Data Availability

The data described in this Data note can be freely and openly accessed on OSF at 10.17605/OSF.IO/AHCYU [9]. Please see Table 1 and reference list for details and links to the data.

## Abbreviations

AMR: adaptive multi-rate
AMR-WB: adaptive multi-rate wideband
AAC: advanced audio coding
AIFF: audio interchange file format
CVSD: continuously variable slope delta modulation
FLAC: free lossless audio codec
GSM-FR: Global System for Mobile Communications Full-Rate
iLBC: internet low bitrate codec
ADPCM: adaptive differential pulse code modulation
MP3: MPEG audio layer-3
PCM: pulse-code modulation
WMA: windows media audio
